# Clinical significance of the internal carotid artery angle in ischemic stroke

**DOI:** 10.1038/s41598-018-37783-1

**Published:** 2019-03-25

**Authors:** Sang-Mi Noh, Hyun Goo Kang

**Affiliations:** 1 Department of Neurology, St. Vincent’s Hospital, School of Medicine, The Catholic University of Korea, Suwon, Republic of Korea; 2 Department of Neurology and Research Institute of Clinical Medicine of Chonbuk National University – Biomedical Research Institute of Chonbuk National University Hospital, Jeonju, South Korea

## Abstract

The carotid artery plays a major role in stroke aetiology and is a good indicator of atherosclerosis. However, the clinical significance of internal carotid artery (ICA) anatomy remains unclear in patients with ischaemic stroke. This study examined the relationship between ICA angle and risk of ischaemic stroke. ICA angles of patients with acute ischaemic stroke were retrospectively compared with those of control patients between March 2014 and July 2014. Controls consisted of those with headaches but without ischaemic stroke. In both groups, ICA angles were measured using Maximum Intensity Projection images from computed tomography angiography, and the relationship between ICA angle and risk of ischaemic stroke was analysed. Of 128 screened patients with acute ischaemic stroke, 27 were enrolled, and 29 with headache were enrolled as controls. No differences were found in baseline characteristics between the two groups, but intracranial stenosis was more frequent in patients with stroke than in controls. Bilateral ICA angles were significantly larger in patients with stroke than in controls. Multiple logistic regression models showed that the right ICA angle was associated with risk of ischaemic stroke. Measuring the ICA angle may help assess the risk of ischaemic stroke.

## Introduction

Ischaemic stroke is one of the leading causes of morbidity and the most important cause of disability in adults. Among the aetiologies of ischaemic stroke, carotid atherosclerosis plays a key role as one third of the leading cause^1–3^. The internal and external carotid arteries originate from the common carotid arteries (CCA), and bifurcations occur between the internal and external carotid arteries (ECA). The bifurcation forms the angles between each set of arteries. Several studies have reported on the clinical significance of the angle and tortuosity of the carotid arteries^4^. Some studies suggested that the internal carotid artery (ICA) angle of origin could be a risk factor of early atherosclerosis^5^. Conversely, another study suggested that the tortuosity in the carotid artery is not related to atherosclerosis^6,7^.

The ICA and the ECA are divided from the CCA, and an angle is formed between the two carotid arteries. These angles lead to local hemodynamic stress in the ICA and carotid bulb, forming atherosclerotic plaque. The ICA angle is defined as the angle between CCA and ICA. Previous studies have reported the effects of the anatomy of the carotid artery on focal atherosclerosis^8^. However, it has not yet been studied whether carotid angle can be evaluated as a vascular risk factor reflecting systemic atherosclerosis. Therefore, we did not evaluate the carotid angle as a risk factor for ipsilateral artery to artery embolic infarction, and compared the carotid angle in the ischaemic stroke and in the general population, excluding the cardioembolic stroke.

## Materials and Methods

### Patients

We conducted the study in the Department of Neurology of St. Vincent’s Hospital from March 2014 to July 2014. We retrospectively investigated the medical records of inpatients with acute ischaemic strokes and those only with headache who underwent magnetic resonance image (MRI) or computed tomography (CT) angiography. We defined patients with acute ischaemic stroke as those who developed clinical symptoms within 7 days and had identifiable acute ischaemic lesion through diffusion-weighted MRI. We excluded the patients who have any risk factor for cardiogenic stroke regardless of atherosclerosis. In addition, those who were identified to have ischaemic stroke because of vasculitis or drug use were excluded from the study. This study aimed to assess the importance of the ICA angle as a systemic vascular risk factor, not to evaluate the role of the ICA angle in patients with ischemic stroke associated with proximal ICA stenosis. Therefore, this study included patients with small vessel-involved ischemic stroke (lacunar infarction) as well as those with large artery-involved ischemic stroke. However, this study excluded patients with cardioembolism, which has a different etiological mechanism, and those with the uncommon cause of stroke, which indicates the occurrence of ischemic stroke due to uncommon factors. The institutional review board of St. Vincent’s Hospital of the Catholic University approved this study (approval number: VC14RISI0254). All procedures were in accordance with the ethical standards of the institutional and national research committees and the Helsinki Declaration.

The control group consisted of patients who claimed to have headaches on the same day as the patients of the experimental group. It would be ideal to use healthy people as a control group. However, the cost of brain CT angiography and consequent radiation exposure made it hard to use them as a control group. Therefore, this study used patients as a control group who underwent brain CT angiography due to a headache and did not have a structural lesion in the test. Furthermore, the control group had neither previous stroke history nor old ischaemic stroke on MRI. If the appropriate control subject could not be assigned on the same day, we selected the control patients from the closest day possible (±3 days). Although, this study is not a matched study based on age, sex, and risk of stroke, patients younger than 45 years old who did not show risk of stroke during examinations were not included. The demographics and vascular risk factors, including hypertension, diabetes, hypercholesterolemia, and smoking, were obtained by reviewing the medical records. Furthermore, patient’s whose ICA angle could not be measured because of proximal ICA or common carotid artery occlusion were excluded. This study was approved by the Institutional Review Board, and informed patient consents were not obtained due to the retrospective observational design of the study. All results in this study were considered as a minimal risk, since authors interpreted patient information through retrospective analysis during diagnosis and all data were encoded to avoid recognition of patients.

### Neuroimaging analysis

All patients with acute ischaemic stroke underwent brain MRI (3.0 Tesla, Ingena, Philips, USA). Diffusion-weighted image, fluid-attenuated inversion recovery (FLAIR), or T2-weighted image was used for imaging analysis. Each patient with headache had several methods of MRI, including FLAIR or T2-weighted image to check the previous ischaemic lesion. CT angiography was used to identify the status of extracranial and intracranial vessels (HD 750, General Electric Scanners, 120-kV tube voltage, 4.5–5 mL/s intravenous contrast speed, and 0.625-mm axial reconstruction thickness) in both.

### Angle measurement

**Figure 1:**
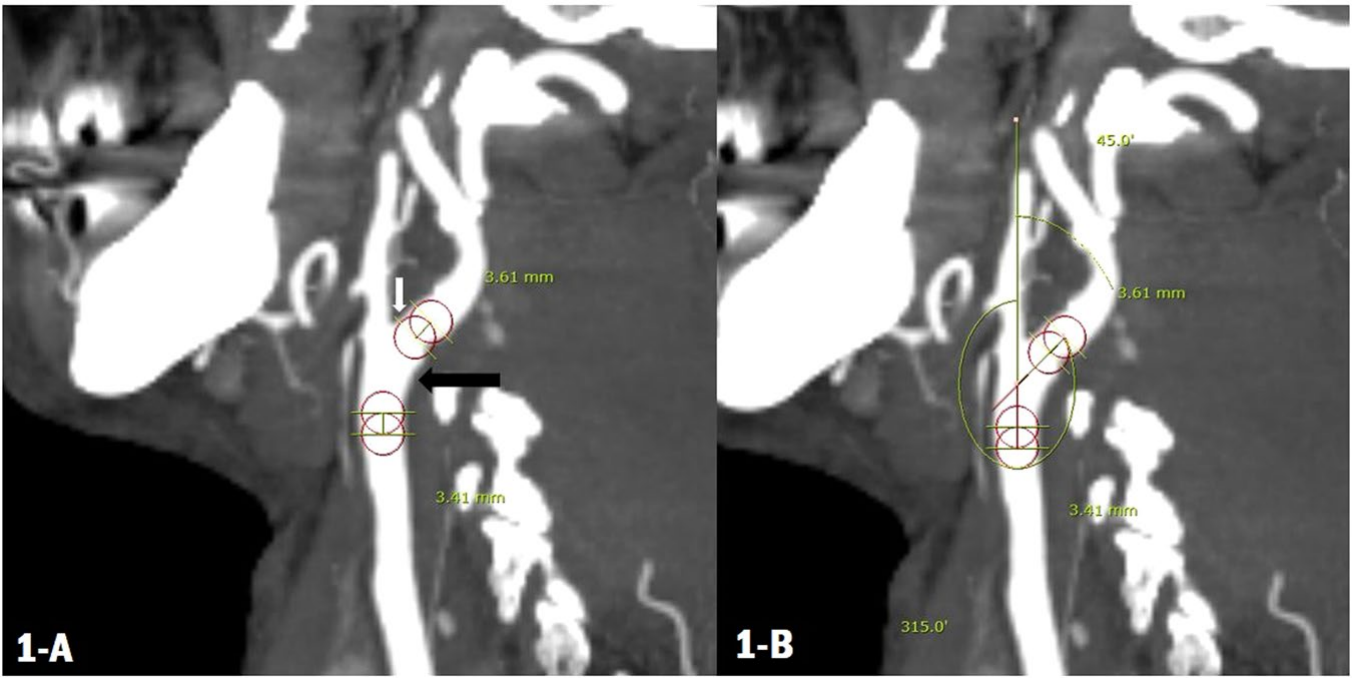
Methods for angle measurement of the internal carotid artery. Two straight lines, one from the common carotid artery to the head and the other from the external carotid artery to the body (**A**), were made by extending the connection of the centre of each circle (**B**). The black arrow indicates the external aspect of the bifurcation, and the white arrow indicates the internal aspect of the bifurcation.

Angle measurement between the common and internal carotid arteries was performed through CT angiography as blind test, and we referred to the previously reported literature^9^. In the maximum intensity projection sagittal image of CT angiography, two circles of maximum radius were placed within 10 mm above the internal aspect of the carotid artery bifurcation. The straight line connecting each centre of the two circles was drawn (Fig. 1), Similarly, two other circles of maximum radius were situated within 10 mm below the external aspect of the carotid artery bifurcation, and each centre of those two circles was connected by another straight line. We measured the angle formed by two lines. With the technique mentioned above, we measured several two-dimensional planes of sagittal image where the carotid bifurcation was the most visible, and the two blinded individuals measured the largest degree of the angle (the largest ICA angle after measuring the angle in multiple Maximum intensity projection (MIP) images). Angle measurement was performed from both the right and left of the ICA.

### Statistical analysis

In the control and experimental groups, we used the patients’ age, sex, vascular risk factors, and brain image to identify if any differences in deviation are observed using the T-test and Chi-square test. We checked for the related variations of ischaemic stroke by using the binominal logistic regression analysis, and we performed multiple logistic regression analysis for the variables with *P* < 0.2. Statistical analyses were performed by using SPSS for Windows version 17.0 (SSS Inc., Chicago, IL, USA). *P*-values less than 0.05 were considered significant.

## Results

During the designated period, 128 patients were admitted with acute ischaemic stroke. Of 128 patients, 101 were excluded because of the following reasons. First, those who had magnetic resonance angiography instead of CT angiography were excluded. Second, those with severe ICA stenosis or calcification that angle measurement was not possible were also excluded. Last, those who had cardiac pacemaker making MRI unavailable were all excluded from the study. Finally, 27 patients and 29 patients with headache were registered in the experimental and control groups, respectively. Thus, 56 patients were included in the study.

The demographics and clinical characteristics of the study population are shown in Table 1. No significant difference was found in the mean age (*P* = 0.468) and sex (*P* = 0.961). Vascular risk factors, including hypertension, diabetes, hypercholesterolemia, and smoking, did not differ between the two groups. More patients with intracranial artery stenosis were observed in ischaemic stroke group: 11 (40.74%) and 7 patients (24.13%) in the ischaemic stroke and control groups, respectively. However, it was not statistically significant (*P* = 0.076).

**Table 1:** Demographics and clinical characteristics.

	Patients (n = 27)	Control (n = 29)	*P-value*
Age, y	65.78 ± 8.46	63.52 ± 11.18	*0*.*468*
Male	16 (59.26%)	17(58.62%)	*0*.*961*
Risk factors			
Hypertension	18 (66.67%)	20 (68.97%)	*0*.*854*
Diabetes	14 (51.85%)	12 (41.38%)	*0*.*432*
Hyperlipidaemia	23 (85192%)	20 (68.97%)	*0*.*151*
Smoking	6 (22.22%)	7 (24.14%)	*0*.*360*
LDL	107.54 ± 40.03	108.48 ± 35.78	*0*.*850*
HDL	44.43 ± 14.71	54.89 ± 52.54	*0*.*337*
TG	151.08 ± 95.19	180.90 ± 136.68	*0*.*440*
Total cholesterol	185.78 ± 47.75)	192.29 ± 47.26	*0*.*783*
Intracranial stenosis	11 (40.74%)	7 (24.13%)	*0*.*076*
Right 1	34.03 ± 8.37	27.20 ± 8.10	*0*.*003*
Left 1	41.78 ± 11.25	32.14 ± 13.62	*0*.*005*
Right 2	34.20 ± 7.49	26.59 ± 8.12	*0*.*001*
Left 2	41.83 ± 10.72	31.43 ± 13.57	*0*.*003*
Mean ICA angle(right)	34.10 ± 7.88	26.89 ± 8.07	*0*.*001*
Mean ICA angle(left)	41.81 ± 10.93	31.77 ± 13.58	*0*.*004*

Results are expressed as patients number (%) or mean (standard deviation). LDL: low-density lipoprotein, HD: high-density lipoprotein, TG: triglycerides, ICA: internal carotid artery.

**Figure 2:**
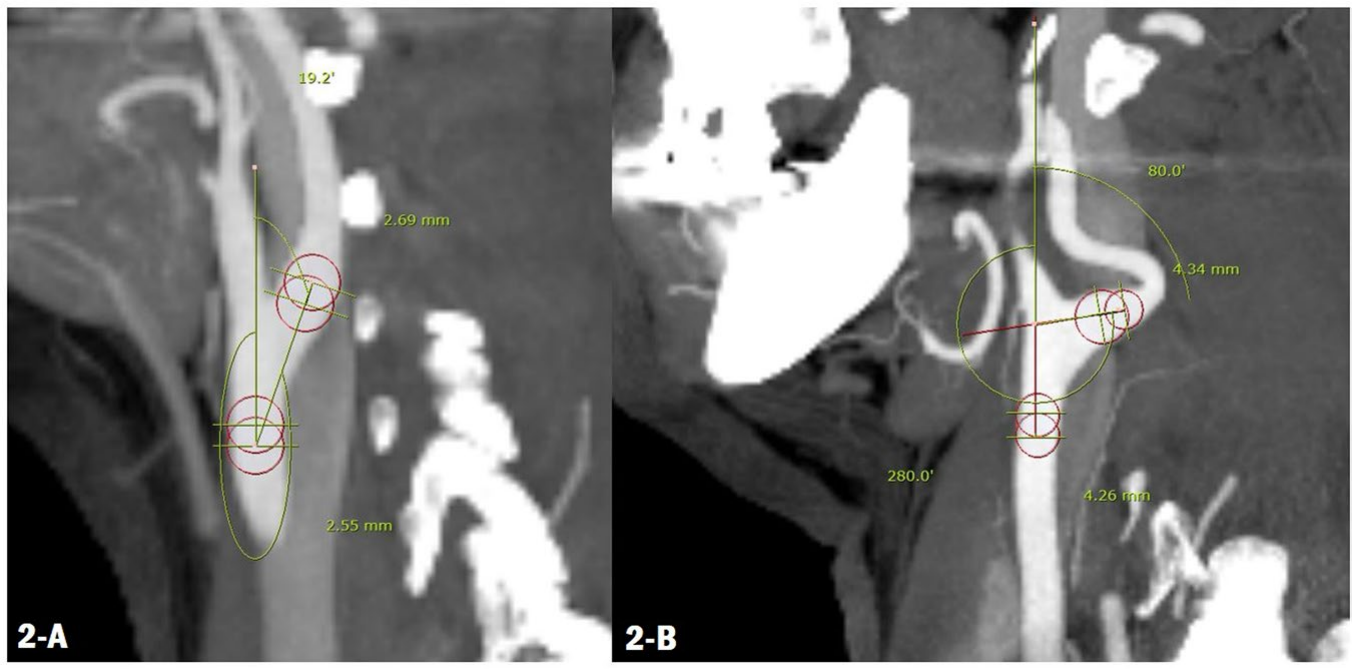
Compare of the ICA angle in control (**A**) vs. stroke patient (**B**).

The angles, measured by two individuals, were significantly larger in patients in the ischaemic stroke group compared with those measured in patients in the control group (*P* < 0.05) (Fig. 2). For the analysis, we used the mean of the angles measured twice, and this analysis also showed the significance (right and left mean, *P* = 0.001 and *P* = 0.004, respectively). Based on a logistic analysis, the mean of value of the ICA angles from both sides was statistically significant (*P* = 0.004 and *P* = 0.008). A correlation was found between dyslipidaemia and intracranial artery stenosis, which did not show statistical significance (Table 2). Multivariable analysis using logistic regression model showed a statistically significant correlation only between the right ICA angle and risk of ischaemic stroke (*P* = 0.003). Intracranial artery stenosis was not statistically significant (P = 0.073), but had a relationship with risk of ischaemic stroke (Table 3).

**Table 2:** Univariate logistic regression analysis for factors of risk of ischaemic stroke.

	Odd ratio	95% confidence interval	*P-value*
Age	1.02	0.97–1.08	*0*.*393*
Male	1.03	0.35–2.98	*0*.*961*
Hypertension	1.11	0.36–3.41	*0*.*854*
Diabetes	0.66	0.23–1.89	*0*.*433*
HbA1C	0.92	0.57–1.49	*0*.*731*
Hyperlipidaemia	0.39	0.10–1.45	*0*.159^*^
TG	0.99	0.99–1.00	*0*.*394*
LDL	0.99	0.98–1.02	*0*.*933*
HDL	0.99	0.97–1.01	*0*.*417*
Total cholesterol	0.99	0.98–1.01	*0*.*644*
Intracranial stenosis	2.16	0.69–6.80	*0*.188^*^
Smoking	1.11	0.32–3.86	*0*.*865*
Mean ICA angle Right	1.13	1.04–1.22	*0*.004^*^
Mean ICA angle Left	1.07	1.02–1.12	*0*.008^*^

^*^Variable which showed P < 0.2 in the univariate analysis were included in the multivariate analysis. LDL: low-density lipoprotein, HD: high-density lipoprotein, TG: triglycerides, ICA: internal carotid artery, HbA1C: glycated haemoglobin.

**Table 3:** Multivariate logistic regression analysis for factors of risk of ischaemic stroke.

	Odd ratio	95% confidence interval	*P-value*
Intracranial stenosis	3.31	0.90–12.28	*0*.*073*
Mean ICA angle Right	1.14	1.05–1.24	*0*.*003*

ICA: internal carotid artery.

## Discussion

Previous several studies on the clinical significance of the tortuosity of the ICAs and the angle between the internal and common carotid arteries showed variable results. We suspect that the inconsistency in those reports is because of the variation in the technology used to measure the angle; for example, measuring devices included carotid artery ultrasonography, CT angiography, transfemoral cerebral angiography, etc. Furthermore, the measurement method also varied from using automated software package or manual measurement. A study based on intima–media thickness and ICA angle checked on the relationship with atherosclerosis, but no study compared the ICA angles between patients with and without ischaemic stroke. We measured the ICA angles from patients with ischaemic stroke and the control group, and found a statistical significance in patients with ischaemic stroke. This result was significant even when no significant differences were found in hypertension, diabetes, age, and other risk factors of ischaemic stroke.

The risk factors for atherosclerosis of extracranial vessels are known to be hypertension, diabetes, coronary artery disease, hypercholesterolemia, smoking, peripheral artery disease, etc.^10–12^. Atherosclerosis of the common carotid artery mainly arises because of these factors, but for the structure of the carotid artery bifurcation, local hemodynamic shear stress works on bulb and becomes a major factor in developing atherosclerosis^13,14^. With regard to the origin of the ICA, which widens naturally, atherosclerotic plaque develops well because of a local hemodynamic effect. Moreover, if stenosis or occlusion develops because of that, ischaemic stroke could happen due to hemodynamic stroke or thrombosis. As mentioned, the ICA anatomy is closely related to ischaemic stroke development, but the method to measure the ICA angle varies because establishing a perfect measuring method is difficult^5,9,15^, and replicating these methods of ICA angle measurements clinically is also difficult. Our research used a relatively easy method of measurement for ICA angles comparing between the ischaemic stroke and control groups, and a statistical significance was found.

However, our study has some limitations. First, we did not use a software package but manually measured the ICA angles, and used two-dimensional plane rather than three-dimensional image reconstruction. However, not all hospitals are able to use these softwares. We used an easy measurement method based on the video from clinical settings; hence, we speculated that this method is more useful. In addition, measurements from two blinded researchers were all statistically significant. Second, we excluded patients who had several carotid artery stenosis or cardiogenic ischaemic stroke, wherein we had difficulty measuring the ICA angles, thus, selection bias is possible.

## Conclusion

The ICA angle in patients with ischaemic stroke resulted to be statistically significant, and this indicates that the ICA angle could be a possible risk factor in ischaemic stroke. Our study is a preliminary study that requires additional larger scale research to test if the ICA angle is indeed an ischaemic stroke risk factor. In addition, the significance of the ICA angle based on ischaemic stroke subtypes to investigate whether the statistical significance is only present in patients with ischaemic stroke with ICA stenosis or is also present in those who have early atherosclerosis needs to be confirmed.
